# Potential role of a navigator gene *NAV3* in colorectal cancer

**DOI:** 10.1038/bjc.2011.553

**Published:** 2011-12-15

**Authors:** E Carlsson, A Ranki, L Sipilä, L Karenko, W M Abdel-Rahman, K Ovaska, L Siggberg, U Aapola, R Ässämäki, V Häyry, K Niiranen, M Helle, S Knuutila, S Hautaniemi, P Peltomäki, K Krohn

**Affiliations:** 1 Department of Dermatology and Allergology, University of Helsinki and Helsinki University Central Hospital, PO Box 160, FI-00029 Helsinki, Finland; 2 Dermagene Oy, Biokatu 8, FI-33520 Tampere, Finland; 3 Department of Medical Genetics, Haartman Institute, University of Helsinki, PO Box 63, FI-00014 Helsinki, Finland; 4 Computational Systems Biology Laboratory, Institute of Biomedicine and Genome-Scale Biology Research Program, University of Helsinki, PO Box 63, FI-00014 Helsinki, Finland; 5 Laboratory of Cytomolecular Genetics, Department of Pathology, Haartman Institute, University of Helsinki, PO Box 63, FI-00014 Helsinki, Finland; 6 Department of Otorhinolaryngology and Helsinki University Central Hospital, University of Helsinki, PO Box 700, FI-00029 Helsinki, Finland; 7 Department of Pathology, Mikkeli Central Hospital, FI-50100 Mikkeli, Finland; 8 Department of Pathology, Centre for Laboratory Medicine, Pirkanmaa Hospital District, PO Box 2000, FI-33521 Tampere, Finland

**Keywords:** colorectal carcinoma, adenoma, *NAV3*, *IL23R*, beta-catenin

## Abstract

**Background:**

The recently described navigator proteins have a multifaceted role in cytoskeletal dynamics. We report here on the relevance of one of them, navigator 3 (NAV3), in colorectal cancer (CRC).

**Methods:**

We analysed changes in chromosome 12 and *NAV3* copy number in CRC/adenoma samples of 59 patients and in 6 CRC cell lines, using fluorescence *in situ* hybridisation, loss of heterozygosity, and array-CGH. *NAV3* target genes were identified by siRNA depletion, expression arrays, and immunohistochemistry.

**Results:**

*NAV3* deletion and chromosome 12 polysomy were detected in 30 and 70% of microsatellite stability (MSS) carcinomas, in 23 and 30% of adenomas and in four of six CRC cell lines. *NAV3* amplification was found in 25% of MSS samples. *NAV3* alterations correlated with lymph node metastasis. In normal colon cells, *NAV3* silencing induced upregulation of interleukin 23 receptor (IL23R) and gonadotropin releasing hormone receptor. In MSS and microsatellite instability tumours, IL23R immunoreactivity correlated with Dukes’ staging and lymph node metastases, whereas nuclear beta-catenin correlated with lymph node metastases only.

**Conclusion:**

*NAV3* copy number changes are frequent in CRC and in adenomas, and upregulation of IL23R, following NAV3 silencing, strongly correlates with Dukes’ staging and lymph node metastases. This suggests that *NAV3* has a role in linking tissue inflammation to cancer development in the colon.

Colorectal cancer (CRC) development from benign precursor lesions, adenomatous polyps, following the accumulation of genetic and epigenetic changes, is one of the best-known examples of multistep carcinogenesis (Vogelstein *et al*, 1988; Fearon and Vogelstein, 1990; Chung, 2000). Recent evidence also suggests that a permissive tissue microenvironment is critical for the growth potential and spread of tumour cells (Kim *et al*, 2006; Reuter *et al*, 2009). Specifically, tumour development is often driven by chronic inflammation. For example, the development of CRC is linked to the presence of inflammatory bowel disease (Xie and Itzkowitz, 2008).

The majority of CRCs display one of two major genomic instability phenotypes, microsatellite instability (MSI) or chromosomal instability with microsatellite stability (MSS; Abdel-Rahman *et al*, 2001). About 85% of CRC exhibit MSS, aneuploidy, and loss of heterozygosity (LOH). Adenomatous polyposis coli (*APC*) and beta-catenin mutations are the most common early molecular aberrations in this phenotype category (Kinzler and Vogelstein, 1996; Polakis, 1997). These mutations lead to aberrant Wnt pathway activation, which is thought to initiate colon adenoma formation. However, second-step activation of *KRAS* is also needed for adenoma progression to carcinoma (Phelps *et al*, 2009). The loss of functional *APC* also induces aneuploidy *in vivo* after a transient tetraploidy stage (Caldwell *et al*, 2007), which may enhance fitness of cells containing broken or rearranged chromosomes.

We have previously shown that chromosome 12q21 aberrations, specifically allelic loss of the neuron navigator 3 (*NAV3*) gene, are associated with several subtypes of cutaneous T-cell lymphoma (CTCL; Karenko *et al*, 2005; Hahtola *et al*, 2008a), CTCL-associated lung cancers (Hahtola *et al*, 2008b), and ca. 25% of cutaneous basal and squamous cell cancers (Maliniemi *et al*, 2011). *NAV3* mutations and copy number changes have thereafter been reported in melanoma (Bleeker *et al*, 2009) and in human glioblastomas, respectively (Nord *et al*, 2009). In addition, *NAV3* was the only differentially expressed gene in adrenal carcinoma relative to adrenocortical adenomas (Soon *et al*, 2009). In genomic landscaping, *NAV3* belongs to the ‘hill type’ candidate cancer gene group, which is commonly mutated in human breast and colon cancer (Wood *et al*, 2007).

We now report that *NAV3* copy number changes are found frequently in MSS type CRC, colon adenomas, and established CRC cell lines. *NAV3* aberrations correlate with chromosome 12 polysomy and with lymph node involvement. To understand the function of *NAV3*, we used specific siRNA gene silencing and expression array analysis to identify relevant *NAV3* target molecules in normal colon cells. These turned out to be gonadotropin releasing hormone receptor (*GnRHR*) and interleukin 23 receptor (*IL23R*). Furthermore, IL23R, GnRHR, and beta-catenin, linked to the GnRH pathway, were upregulated in patient-derived tumour tissue samples and their expression correlated to several unfavourable clinical parameters. Our findings suggest that *NAV3* copy number changes promote early carcinogenesis by upregulating *IL23R* and *GnRHR* expression, contributing to a microenvironment of inflammation, and providing malignant cells with a growth advantage.

## Materials and methods

### Tissue samples

Surgical biopsy samples were collected from 59 patients (61 CRC and 10 adenoma samples) who underwent surgical resection of CRC tumours at Mikkeli Central Hospital, Mikkeli, Finland. The study was approved by the Ethical Review Board of Mikkeli Central Hospital and by The National Authority for Medicolegal Affairs, Helsinki, Finland. Histology of formalin-fixed, paraffin-embedded tissue samples was assessed by an experienced pathologist (MH), and tumours, adenomas, or normal mucosa were microdissected after a second analysis by two experienced pathologists (WA-R, KK) to obtain either pure normal epithelium or at least 50% carcinoma or adenoma tissue. Paraffin-embedded sections were cut at 50-*μ*m thickness, nuclei were isolated for fluorescence *in situ* hybridisation (FISH) analysis, and DNA was purified for LOH analysis following standard protocols (Hyytinen *et al*, 1994; Isola *et al*, 1994). All adenomas were MSS, whereas 14 of the 56 carcinomas had high-degree MSI (MSI analysis in Supplementary Online Materials, Method 1). The following parameters were recorded from each patient: tumour grade and stage by Dukes and TNM classification as defined by the American Cancer Society (Oklahoma City, OK, USA), presence of lymph node metastasis, follow-up time, and clinical outcome.

### Cells and cell lines

CRL-1541 (CCD-112CoN) and CRL-1539 (CCD-33Co; ATCC, Manassas, VA, USA) normal colon cells, and CRC cell lines CCL-228 (SW480), CCL-230 (SW403), CCL-248 (T84), CRL2577 (RKO), LIM1215, and HCA7 (ATCC), were grown as instructed by ATCC. All assays on CRL-1541 and CRL-1539 cell were performed on cells provided by ATCC that were grown for no more than six passages. Colon carcinoma cell lines CCL-228, CCL-230, CCL-248, and CRL-2577 were also provided by ATCC and grown for a maximum of 20 passages before use. Metaphase preparates of LIM1215 and HCA7 cells were provided by professor Päivi Peltomäki, and cells have previously been described in detail (Abdel-Rahman *et al*, 2001; Joensuu *et al*, 2008). RKO, LIM1215, and HCA7 are mismatch repair deficient and have MSI.

### Fluorescence *in situ* hybridisation

*NAV3*-specific FISH was performed on nuclei isolated from tissue samples (Hyytinen *et al*, 1994; Isola *et al*, 1994) and on metaphase chromosomes of colon carcinoma cell lines. Bacterial artificial chromosome clones containing *NAV3* DNA (RP11-36P3 and RP11-136F16; Research Genetics Inc., Huntsville, AL, USA) and the chromosome 12 centromere probe (pA12H8; ATCC) were labelled with Alexa 594-5-dUTP and Alexa 488-5-dUTP (Invitrogen, Carlsbad, CA, USA), respectively, for tissue samples, or with digoxigenin and biotin, respectively, for cell lines. Detailed methods for probe labelling, slide preparation, and arm-specific MFISH are provided in Supplementary Online Materials, Method 2. The FISH results of the tissue samples were analysed by two blinded, independent researchers. Results are reported as the percentage of abnormal nuclei in 200 total nuclei as previously described (Ranki *et al*, 2011). For each cell line, 9–47 metaphases were analysed for *NAV3* and centromere 12, whereas 5–11 metaphases were analysed for arm MFISH.

### *NAV3* LOH analysis using microsatellite markers and single-nucleotide primer ex tension

*NAV3* LOH assays were performed as previously described (Hahtola *et al*, 2008b). In addition, the A/G polymorphism (rs1852464) within exon 19 of the *NAV3* gene, which exhibits up to 0.493 heterozygosity in Caucasians/Europeans, was used in the single-nucleotide primer extension reaction. Detailed methods are provided in Supplementary Online Materials, Method 3. Tumour samples were defined as showing LOH, if one allele had 40% more or less rs1852464 signal than matched normal tissue. In the event of constitutional homozygosity, LOH was assessed using flanking microsatellite markers (Cleton-Jansen *et al*, 2001).

### Array CGH

DNA was extracted from 50-*μ*m paraffin-embedded tissue sections by standard protocols. Reference DNA was extracted from blood pooled at the Finnish Red Cross (Helsinki, Finland) from four healthy males and females after informed consent. DNA was then digested, labelled, and hybridised to a 244-K oligonucleotide array according to the manufacturer's protocol (Agilent Technologies, Santa Clara, CA, USA). Samples were scanned with a DNA microarray scanner and analysed using Feature Extraction v. 9.5.3.1 and CGH Analytics software v. 3.5.14 (Agilent Technologies). Analysis was performed using the *Z*-score and a 1-Mb moving average window. Log2-values under ±0.4 were not considered aberrant. Three colon carcinoma cell lines and two colon carcinoma tumour samples were analysed.

### *NAV3* gene silencing *in vitro*

The *NAV3* gene was silenced with pooled siRNA oligonucleotides (On-Target SMART pool, Dharmacon, Chicago, IL, USA) as instructed by the producer. The CRL-1541 and CRL-1539 cell lines were transfected with 200 pmol *NAV3* pooled siRNA or scrambled control siRNA (Dharmacon), using Dharmafect1 transfection reagent (Dharmacon; for details see Supplementary Online Materials, Method 4). Efficient knockdown was confirmed by qPCR and microarrays (as shown in Supplementary Figure 1). Efficient *NAV3*-silencing and upregulation of *IL23R* and *GnRHR* were confirmed by LightCycler qPCR (Roche, Basel, Switzerland) using *TBP* (tata box binding protein) as reference gene for normalisation (primers used are given in Supplementary Table 1). As according to the manufacturer, the optimal timepoint for RNA knockdown assessment is 24–48 h, the following post-transfection RNA samples were analysed on Agilent 4 × 44 K microarrays: CRL-1541 (6 and 48 h) and CRL-1539 (6 and 24 h).

### Gene expression microarrays and microarray analysis

To identify genes regulated by *NAV3*, changes in gene expression profiles induced with *NAV3*-targeted RNAi were analysed with Agilent 4 × 44 K dual-color microarrays (Biochip Center, Biomedicum Helsinki, http://www.helsinki.fi/biochipcenter/). RNA was purified with RNeasy Micro or Mini Kit (Qiagen, Hilden, Germany) and stored at −70°C. Total RNA obtained from the *NAV3*-silenced cells was hybridised with the corresponding time point samples from the same cells transfected with scrambled oligonucleotides. Before hybridisation, RNA sample quality was assessed with a 2100 Bioanalyzer (Agilent Technologies). The microarray data from the four normal colon cell lines were analysed using the Anduril bioinformatics framework (Ovaska *et al*, 2010). Probe intensities were background corrected and normalised with LOWESS, using Agilent Feature Extractor 9.1.3.1. Genes with fold change >2 or <0.5 in all samples were considered as differentially expressed. Stringent quality control, which removes probes not flagged as above background by Agilent Feature Extractor, resulted in only one differentially expressed gene in addition to NAV3. Accordingly, the quality filtering criteria were loosened to include genes with low signal intensity. Probes whose sequence could not be uniquely mapped to Ensembl v.56 transcripts were discarded (Gertz *et al*, 2009).

### Immunohistochemistry on tissue microarrays

IL-23R and beta-catenin expression was studied by immunohistochemistry of tissue microarrays prepared from paraffin-embedded colon biopsies of the above patients. From each patient, paired samples from the histologically normal colon and two samples from the colon tumour were included. Also, 10 paired samples of an adenomatous lesion and another sample from the normal colon were included. Altogether, 57 patients, 43 MSS tumour samples, 14 MSI tumour samples, 14 adenoma samples, and 57 corresponding normal colon samples were included. Detail methods are available in Supplementary Online Materials, Method 5. For statistical analysis, samples were scored as follows: for IL-23R immunoreactivity: no staining (score 0), weak positive staining (score 1), clear positive staining (score 2), strong positive staining (score 3); for beta-catenin: no staining (score 0), cell membrane staining (score 1), cytoplasmic staining (score 2), only nuclear staining in the majority of tumour cells (score 3).

### Statistical analysis of tissue samples

Correlations between categorical variables studied were analysed using the *χ*^2^-test, or when not valid, the Fisher's exact test. Also, the following parameters were included in the statistical analysis: tumour grade and stage by Dukes and TNM classification, presence of lymph node metastasis, follow-up time, and clinical outcome. Survival analyses were performed using death caused by colon cancer as the primary endpoint. Follow-up times were obtained from Statistics Finland (Helsinki, Finland). SPSS version 15.0 software (SPSS Inc., Chicago, IL, USA) was used.

## Results

### *NAV3* copy number changes are found in CRC and colorectal adenomas

**Figure 1:**
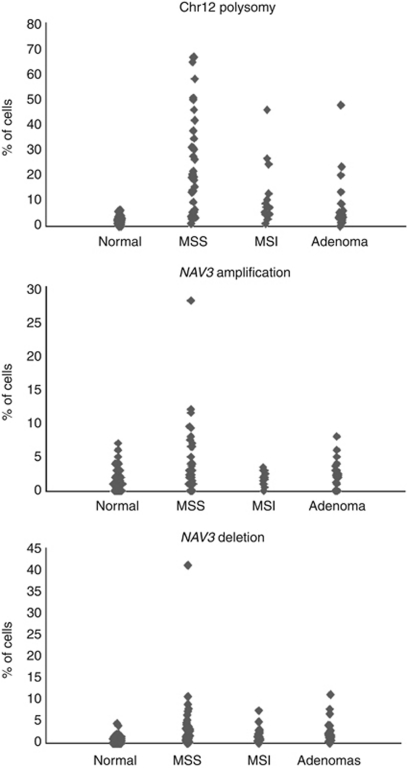
(**A**) Chromosome 12 polysomy, (**B**) *NAV3* amplification, and (**C**) *NAV3* deletion in normal colon, MSS, and MSI colon carcinoma and in colon adenoma samples. Each bullet represents one sample and 200 cells were counted per sample.

Cells with *NAV3* copy number changes were detected in 40% of MSS-type colorectal carcinoma samples as follows: a mixed cell population of those with *NAV3* deletion and those with *NAV3* amplification was detected in 15% of samples, *NAV3* deletion alone in 15%, and low levels of *NAV3* amplification (three to five copies) alone in 10%. Cells with *NAV3* deletion were also detected in 12.5% of MSI-type samples and 23% of adenoma samples. In addition, cells with chromosome 12 polysomy, most often three or five copies, were detected in 70% of MSS-type colorectal carcinoma samples, 50% of MSI-type samples, and 31% of adenoma samples (Figure 1). *NAV3* copy number changes were confirmed by LOH assay, and LOH was detected in 21% of MSS carcinomas and in 18% of adenoma samples overall (marker-specific frequencies for LOH are given in Table 1).

**Figure 2:**
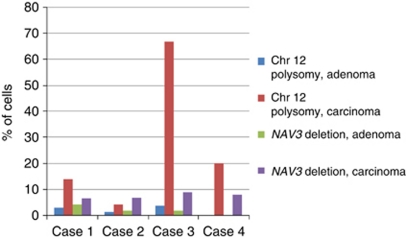
Amount of chromosome 12 polysomic and NAV3-deleted cells in adenoma and carcinoma samples from the same patients.

Comparison of *NAV3* copy numbers between adenoma and carcinoma samples from the same intestinal resection samples of a given patient revealed that *NAV3* deletion also was frequently detectable at the adenoma stage, however at lower frequency than in the corresponding carcinoma sample (Figure 2).

### *NAV3* aberrations associate with chromosome 12 polysomy and lymph node metastasis

*NAV3* copy number changes correlated significantly with chromosome 12 polysomy and with lymph node metastasis (Table 2). No statistically significant correlations were found between *NAV3* status, Dukes' stage, tumour grade, or patient history of other malignancies. For survival analysis, this patient cohort was not appropriate, as the follow-up time was short and the homogeneity of treatment could not be guaranteed.

### CRC cell lines show *NAV3* copy number changes or translocations

**Figure 3:**
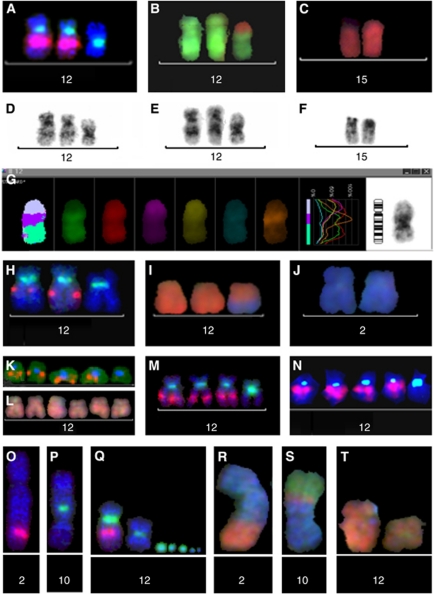
Moleculocytogenetic specification of chromosome 12 and NAV3 aberrations in colon cancer cell lines. The aberrant cells of lines CCL-230 (**A**–**G**) and CRL-2577 (**H**–**J**) most commonly showed three copies of chromosome 12 (**A**, **H**: centromere 12 green), with loss of NAV3 by specific bacterial artificial chromosome (BAC)-probes in one of them (**A**: RP11-36P3 red, **H**: RP11-136F16 red). In the cell line CCL-230, a translocation from chromosome 15 (**B**, **C**: red; **G**: wine-red in arm-MFISH) to chromosome 12 (**B** and **G**: green) was specified to 12p (**G**: orange), and 12q showed a deletion (**D**–**F**: shortened q-arm and change of the inverted DAPI banding pattern). In the cell line CRL-2577, an unbalanced translocation between chromosomes 12 (**I**: red, MFISH) and 2 (**I**, **J**: blue, MFISH) abolished the *NAV3* gene. The line CLL-248 mostly showed five copies of chromosome 12, one of them missing NAV3 (**K**: centromere 12 blue, BAC RP11-36P3, orange), but no translocations in arm-MFISH (**L**). The deletion extended to the regions defined by YAC probes 825F9 (**M**) and 885G4 (**N**). In the line CLL-228, (**O**–**T**) the NAV3- specific signal (RP11-36P3, red) was seen in one abnormal chromosome (**O**) and in the normal chromosome 12 (Sub-figure **Q**), but not in two other chromosomes with centromere 12 (Sub-figures **P**, **Q**: centromere 12 green), nor in the minute chromosomes with centromere 12 material (Sub-figure **Q**: green). The corresponding aberrant chromosomes by arm-MFISH (**R**–**T**) are t(2;12) (**R**: blue; orange), der(10)t(10;12; 3) (**S**: blue; orange; green) and i(12)(p) (**T**: orange, the aberrant chromosome is on the right, a normal chromosome 12 is on the left). YAC-and BAC probes as published previously (12).

Six established CRC cell lines and two normal colon cell lines were analysed with *NAV3*-specific FISH (Supplementary Table 2). The normal colon cells CRL-1541 and 92% of CRL-1539 cells showed no aberrant *NAV*3 or chromosome 12 centromere signals, and the remaining 8% of CRL-1539 cells were tetrasomic nuclei. CCL-230 (>90%), CRL-2577 (>40%), and CCL-248 (>90%) cells displayed NAV3 deletion. Cells of the near-diploid line CCL-228 typically showed one normal chromosome 12, two abnormal chromosomes missing *NAV3*, and one abnormal chromosome with *NAV3*-signal, but no chromosome 12 centromere signal. A translocation of *NAV3* to another chromosome, interpreted as t(2;12) by arm MFISH, was observed in all metaphases, except one (Figure 3 and Supplementary Table 2).

### Array-CGH analysis of tumour tissue and CRC cell lines

Array-CGH studies were performed on two patient samples and on three established CRC cell lines. Array-CGH data demonstrated a deletion in 12q21, spanning the *NAV3* locus in one patient sample, thus confirming the FISH results (patient sample had 41% *NAV3* deleted cells by FISH; Supplementary Figure 2). However, the other patient sample showed normal results by this analysis, probably due to an insufficient proportional number of *NAV3* aberrant cells in the sample (28% of cells showing amplified *NAV3* signals by FISH). Array-CGH analysis of colon carcinoma cell lines showing *NAV3* loss by FISH revealed major alterations in chromosome 12, as well as in other chromosomes, as was expected for cultured cancer cells. In the CLL-230 line, a wide deletion spanning the *NAV3* locus was detected in 12q. This deletion was not detected in the other two cell lines (CLL-248 and CLL-228), which instead had amplifications of other parts of the chromosome.

### *NAV3* gene silencing results in the upregulation of GnRHR and IL23R in normal colon cell lines and corresponding association is seen in CRC cell lines with *NAV3* deletions

To identify *in vivo* relevant target genes of NAV3, we studied the gene expression profiles of *NAV3*-silenced normal colon cells (with normal *NAV3* gene copy numbers). On the basis of the microarray data, we selected two membrane receptors, *GnRHR* (fold change >14 in all cell lines) and *IL23R* (fold change >4 in all cell lines), from the list of 55 putative differentially expressed genes (Supplementary Table 3) for further analysis. Both *GnRHR* and *IL23R* receptors are involved in carcinogenesis by activating GnRHR and Jak-STAT pathways, respectively. These genes were also the only ones directly connected to downstream signalling pathways, and thus, of special interest. Upregulations were confirmed with qPCR when NAV3-silenced cells (CRL-1541, 48 h post-transfection) were compared with control cells showing two-fold increase in GnRHR and four-fold increase in IL-23R mRNA levels. In addition, when studying *NAV3* expression with qPCR in a CRC cell line with *NAV3* deletions, decreased *NAV3* mRNA levels were observed compared with the normal colon cells (Supplementary Table 4). In line with the siRNA gene silencing results, the *IL23R* mRNA level was increased in the CRC cell line CCL-248 with 90% *NAV3* deletions compared with the normal colon epithelial cells (Supplementary Table 3).

### Expression of IL-23R in MSS tumours correlated with Dukes' staging and lymph node metastasis

**Figure 4:**
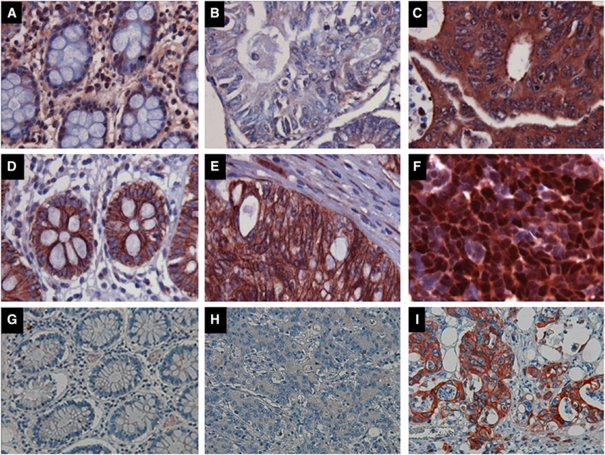
Expression and cellular localisation of IL23R and beta-catenin in human colon and CRC. Representative photomicrographs of immunostaining for IL23R (**A**) and beta-catenin (**D**) in normal colon tissue. Upregulated IL23R immunoreactivity was observed in CRC samples with *NAV3* deletion (**C**, strongest grade 3 immunostaining), whereas cancers without *NAV* aberration showed no IL23R staining (**B**). Both samples (**B** and **C**) had 20% tumour cells with chromosome 12 polysomy. A CRC sample with no *NAV*3 deletion showed normal membranous pattern of beta-catenin staining (**E**) whereas a CRC sample with *NAV3* deletion in 9% of the cells showed mainly strong nuclear localisation of beta-catenin (**F**). NAV3 immunostaining of normal colon (**G**), of CRC with *NAV3* deletion (**H**) and an area of a CRC tumour with amplified *NAV3* (**I**).

Immunohistochemical detection of beta-catenin (linked to the GnRH pathway) (Salisbury *et al*, 2008) and IL-23R expression was performed on a tissue microarray of 43 MSS (including 8 matched adenoma samples) and 14 MSI (matched tumour and normal colon epithelium) patient samples. In all but three normal colon samples, beta-catenin staining was membranous and IL-23R expression was undetectable or weak (grade 1). Samples with allelic *NAV3* deletions frequently showed upregulated IL-23R expression, ranging from weak positive staining to strong positive staining (Figure 4). Beta-catenin is known to localise to the cell membrane in normal epithelial tissue, but relocalise to the nucleus and cytoplasm in carcinoma tissue (MacDonald *et al*, 2009). Duplicate analysis of MSS tumour samples revealed only nuclear beta-catenin in 10 of 43 samples and upregulation of IL-23R-immunoreactivity in 27 of 43 samples. The nuclear beta-catenin expression correlated with lymph node metastasis (Table 2). Upregulated IL-23R immunoreactivity correlated with Dukes' staging and lymph node metastases (Table 2).

For 11 MSS-type CRC patients, it was possible to compare the IL-23R, beta-catenin, and *NAV3* FISH results in matched adenoma and tumour samples. *NAV3* copy number changes and elevated IL-23R expression (moderate or strong immunostaining), and/or nuclear beta-catenin were present in three of the adenoma samples. Elevated IL-23R expression alone was present in two additional adenomas (data not shown). In the corresponding tumour samples, the finding was similar, except that two samples had no *NAV3* aberrations despite the presence of upregulated IL-23R expression. In both cases, the corresponding adenoma lesion had chromosome 12 polysomy. Because of the small number of adenoma samples in these surgical resections, it was not possible to assess correlation between the *NAV3* copy number changes, IL-23R and beta-catenin expression, biological behaviour, and prognostic features of the disease.

## Discussion

The current paper describes copy number changes in chromosome 12 centromere, and in the *NAV3* gene, located in 12q21, in a substantial number of cases of colorectal carcinomas or colon adenomas. Furthermore, the functional consequences of *NAV3* deletion were characterised with siRNA silencing experiments. The *NAV3* copy number changes correlated with chromosome 12 polysomy, as well as with the occurrence of lymph node metastases in the patients.

An obvious question to be addressed is whether the observed NAV3 aberrations would just reflect copy number changes in larger areas of chromosome 12. The LOH method used in this study, would not by itself have excluded such an interpretation as changes close to the location of the *NAV3* gene (for example, the cancer implicated gene *E2F7* (Endo-Munoz *et al*, 2009)) would also have shown loss of heterogeneity in this assay. However, the *E2F7* gene sequence is outside the FISH probe used to enumerate NAV3 copy numbers in the studied samples. Also, the FISH probe does not cover the 12q13.13 locus recently identified as an additional CRC susceptibility locus (Houlston *et al*, 2010).

Another question of importance is whether the observed NAV3 aberrations would be causative factors (‘driver alterations’) in cancer formation or spread, or whether they would represent secondary changes. Generally, features that would support a ‘driver’ action of a given gene or alteration include a high frequency of alterations, several alternative types of changes that can inactivate/activate a gene, involvement of many different types of tumours, being part of a relevant biological pathway, and experimental evidence of important functional consequences (Boland *et al*, 1998; Leary *et al*, 2008; Kalari and Pfeifer, 2010). Most of these general characteristics of a causative factor apply to NAV3, as demonstrated by others and us. Frequent *NAV3* copy number changes were identified in CTCL (Karenko *et al*, 2005) and in two other epithelial cancers: basocellular and squamocellular carcinomas of the skin (Maliniemi *et al*, 2011). In CTCL, deletion of *NAV3* correlated with poor prognosis or with poor response to therapy (Ranki *et al*, 2011). Earlier studies by Wood *et al* (2007) show that *NAV3* is a ‘hill-type’ cancer gene, and that in CRC, there are several different point mutations in this gene. In contrast to APC mutations that are all truncating and would lead to inactivation of the gene, the NAV3 point mutations are of missense type and could thus theoretically lead both to increased or decreased activity. NAV3 is a helicase and it was recently shown that a similar gene, the helicase-like transcription factor (*HLTF*), can have a dual-type of activity, behaving both as an oncogene and as a tumour suppressor (Debauve *et al*, 2008). Finally, NAV3 silencing experiments described in the present investigation provide evidence that a proper function of NAV3 is critical for central biological processes relevant for tumourigenesis.

To further characterise the biological effect of NAV3 deletion, NAV3 expression was silenced with siRNA in normal colon cells, with normal NAV3 copy numbers. In these studies, the upregulation of two signalling pathways, the Jak-STAT and GnRH pathways (regulated by IL-23R and GnRHR, respectively) were seen.

Strong IL23R expression and the translocation of beta-catenin, regulated by the GnRH/GnRHR pathway, were seen in some of the patient samples and correlated with signs of poor prognosis, namely Dukes' staging and the presence of lymph node metastasis. Also, IL23R mRNA level was upregulated in a colon carcinoma cell line showing up to 90% cells with NAV3 deletion.

The GnRHR/GnRH signalling pathway in extrapituitary tissues, and in a variety of tumours, is thought to be related with non-classical GnRHR signalling pathways, including the regulation of several proteins associated with cell proliferation and cell motility (reviewed in Aguilar-Rojas and Huerta-Reyes (2009)). GnRHR is known to transmit signals via beta-catenin (Salisbury *et al*, 2008; Gardner and Pawson, 2009), the key signalling molecule of the canonical Wnt pathway (Gordon and Nusse, 2006), whereas growth factors and inflammatory factors have been suggested to activate the Wnt pathway in CRC to stimulate the mobility of tumour cells (DeNardo *et al*, 2008). Our observation of GnRHR upregulation as a consequence of *NAV3* silencing in normal human colon cells and, correspondingly, the correlation of nuclear beta-catenin expression with lymph node metastases in the clinical CRC samples substantiates the role of NAV3 as one of such activating factors.

The upregulation of the *IL23R* in CRC is of interest in view of previous reports on significantly elevated mucosal levels of *IL23R* mRNA in Crohn's disease (Kugathasan *et al*, 2007; Holtta *et al*, 2008) and more recent reports linking an *IL23R* polymorphism to the development of inflammatory bowel diseases, typically with an increased risk of CRC (Lakatos *et al*, 2008; Einarsdottir *et al*, 2009; Silverberg *et al*, 2009; Yang *et al*, 2009). Thus, future prospective studies will need to verify whether a smouldering inflammation with IL-23 secreted by activated inflammatory cells (Brand, 2009) in the intestinal microenvironment would contribute to the development of sporadic CRC as well.

In conclusion, *NAV3* copy number changes may provide at least two growth advantages to a subpopulation of tumour cells. Tumour cells with NAV3 aberrations and abnormal localisation of beta-catenin would become less susceptible to growth control mechanisms by surrounding cells, whereas they also become more susceptible to growth promotion by tissue inflammatory signals, including IL23. Our demonstration that *NAV3* aberrations are linked to inflammation and cell proliferation pathways, and finally to lymph node metastasis, may thus identify the cell population responsible for the spread of the initially local tumour.

## Figures and Tables

**Table 1:** *NAV3* LOH results for each marker

	**D12S1684**	**D12S326**	**SNuPE@rs1852464**	**D12S1708**
CRC, MSS	8/37 (22%)	10/33 (30%)	4/23 (17%)^a^	5/29 (17%)
CRC, MSI	0/3	0/2	0/7^a^	0/4
Adenoma	1/21 (5%)	2/19 (11%)	0/8^a^	2/16 (13%)

Abbreviations: CRC=colorectal cancer; LOH=loss of heterozygosity; MSI=microsatellite instability; NAV3=navigator 3; SNuPE=single-nucleotide primer extension.

a LOH frequencies were based on informative cases. SNuPE test was uninformative due to constitutional homozygosity in five carcinomas and in all adenomas that showed LOH by chromosome 12 microsatellites examined.

**Table 2:** Correlations between *NAV3* copy number, chromosome 12 polysomy, tumour grade, Dukes' stage, and metastasis

	**Chromosome 12 polysomy**	**Tumour grade**	**Dukes grade**	**Lymph node metastases**
*NAV3* copy number change	*P*=0.005	ns	ns	*P*=0.045
	*n*=54	*n*=51	*n*=51	*n*=51
Chromosome 12 polysomy		*P*=ns	ns	*P*=0.023
		*n*=51	*P*=51	*n*=51
Nuclear beta-catenin expression	ns	ns	ns	*P*=0.048
	*n*=47	*n*=49	*n*=49	*n*=49
Upregulated IL-23R immunoreactivity	ns	ns	*P*<0.001	*P*<0.001^a^
	*n*=46	*n*=47	*n*=47	*n*=47

Abbreviations: IL23R=interleukin 23 receptor; NAV3=navigator 3; ns=not statistically significant.

Fisher's exact test was used if not otherwise indicated.

a *χ*^2^-test.
