# Supplementary material for: Potential role of a navigator gene NAV3 in colorectal cancer
Source: Br J Cancer. 2011 Dec 15;106(3):517–24. doi: 10.1038/bjc.2011.553 (PMC3273355; doi:10.1038/bjc.2011.553)
Supplement: Supplementary Figures 1 and 2 [file bjc2011553x1.doc]

Supplementary online material, Figures


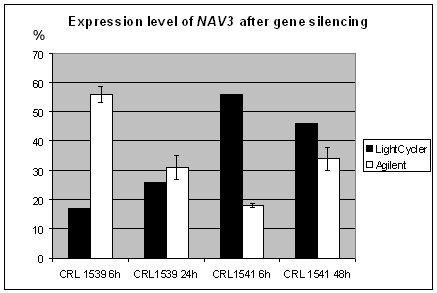


**Figure 1.Confirmation of *NAV3* downrefulation in siRNA silenced CRL cell lines.** The mRNA level were assessed by LightCycler qPCR and Agilent 4 x 44 K microarrays. The mRNA for both assays originated from the same sample at each time point. Black bars indicate the expression value in qPCR, wheras the white bars show values from the microarrays.


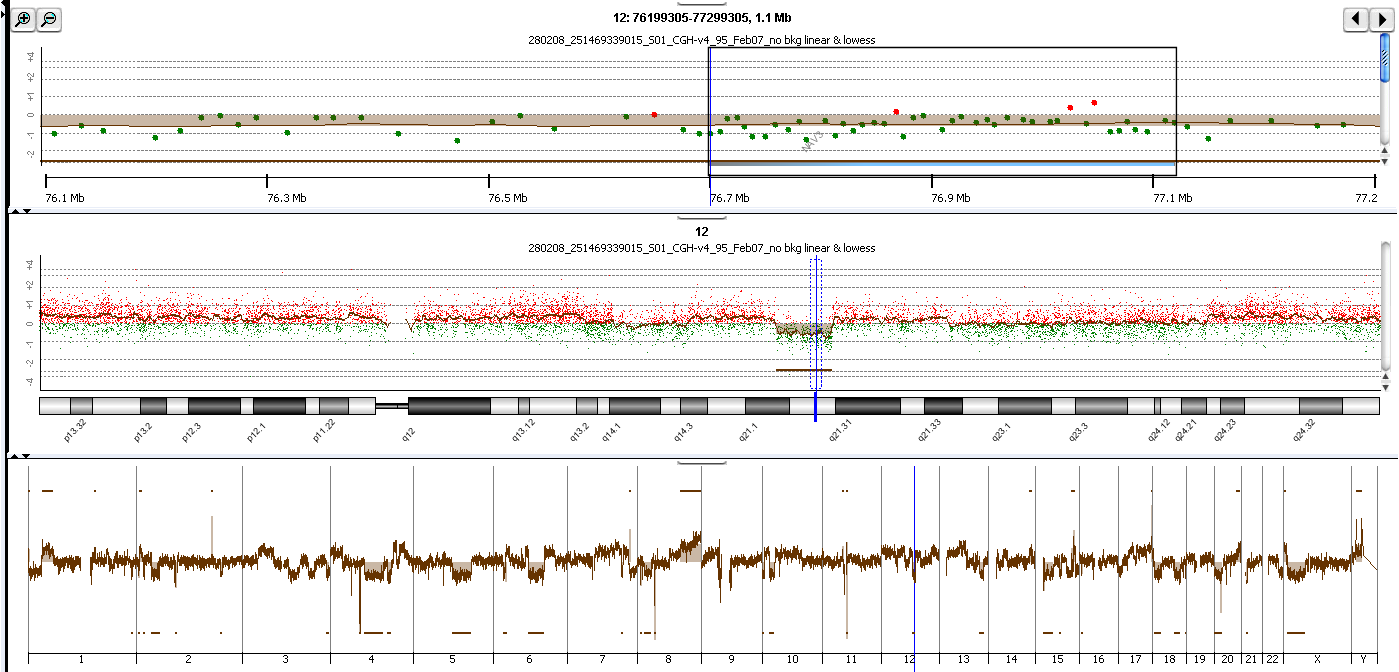


**Figure 2**. Loss of *NAV3* in a colon carcinoma tumor sample, as detected by array CGH. Views from bottom: genome overview; chromosome 12 overview; gene level where *NAV3* probes marked by square box. The majority of *NAV3* probes lie below the zero-line, indicating a loss.
