# Supplementary material for: Potential role of a navigator gene NAV3 in colorectal cancer
Source: Br J Cancer. 2011 Dec 15;106(3):517–24. doi: 10.1038/bjc.2011.553 (PMC3273355; doi:10.1038/bjc.2011.553)
Supplement: Supplementary Tables 1–4 [file bjc2011553x2.doc]

Supplementary online material, tables

Table 1. PCR primers used in qPCR

| Gene | fwd primer | rew primer |
| --- | --- | --- |
| NAV3 | ATCCATGGAGCTCAGCAA | TTGGCTGCTTCTTGGAGTTT |
| GnRHR | AAGAGCACGGCT GAAGACTC | GCATGGGTTTAAAAAGGCAA |
| IL23R | CGCAAAACTCGCTATTCGACA | ATGGCTTCCCTCAGGCAGA |
|  |  |  |

Table 2**.** *NAV3* or centromere 12 aberrations in CRC cell lines

| **Cell line** | **Type** | **Most common ploidy level, frequency, chromosome number range** | ***NAV3* FISH** | **Arm MFISH, MFISH, or YAC** |
| --- | --- | --- | --- | --- |
| CCL-230 | CIN | near-triploid,11/15, range 34 to 212 | Loss of N*AV3* in 14/15 metaphases | Unbalanced der(12)t(12;15)(p13?;q?)  del(12)(q15or 21) in 6/6 metaphases |
| CCL-248 | CIN | near-diploid, 7/10,  range 75 to 114 | Loss of *NAV3* in 9/10 metaphases | Del(12)(q?q?) in 5/5 metaphases in arm MFISH, deletion by YAC FISH in 10/10 metaphases, see figure |
| CCL-228 | CIN | near-diploid, 6/10,  range 34-183 | Fragmentation or amplification of centromere 12 in 10/10 metaphases | der(2)t(2;12)[5], der(10)t(3;12;10)[3], i(12)(p)[4], inv(12)(p12q12or13) del(12)(p?)del(12)(q?)[2]  in six metaphases |
| CRL-2577 | MSI | near-diploid, 34/47, range 15 to 90 | Loss of *NAV3* in 21/47 metaphases studied | Unbalanced der(12)t(2;12) in 6/11 metaphases |

Cell lines LIM1215 (MIN) and HCA7 (MIN) showed as many centromere 12 as *NAV3*-signals (27/27 and 27/29 metaphases studied, respectively), as even the clonal 12q+ chromosome in HCA7 observed previously (7) showed *NAV3* signal in the present study.

Table 3: genes differentially expressed in NAV3 silencing experiments.

| **GeneName** | **Expression (median)** | **CRL-1539 6h** | **CRL-1539 24h** | **CRL-1541 6h** | **CRL-1541 48h** | **Description** |
| --- | --- | --- | --- | --- | --- | --- |
| C12orf42 | 42,55 | 44,22 | 40,94 | 17,63 | 47,25 | chromosome 12 open reading frame 42 [Source:HGNC Symbol;Acc:24729] |
| ARL11 | 20,80 | 30,52 | 22,89 | 6,83 | 18,90 | ADP-ribosylation factor-like 11 [Source:HGNC Symbol;Acc:24046] |
| SDR16C5 | 16,99 | 20,70 | 19,53 | 7,72 | 14,77 | short chain dehydrogenase/reductase family 16C, member 5 [Source:HGNC Symbol;Acc:30311] |
| GNRHR | 16,51 | 32,75 | 15,66 | 14,80 | 17,41 | gonadotropin-releasing hormone receptor [Source:HGNC Symbol;Acc:4421] |
| RP11-91K11.2 | 14,41 | 27,62 | 13,36 | 15,54 | 4,13 | [undefined] |
| GAPT | 12,44 | 23,65 | 9,27 | 7,04 | 16,71 | GRB2-binding adaptor protein, transmembrane [Source:HGNC Symbol;Acc:26588] |
| AQP10 | 11,50 | 15,23 | 16,26 | 8,68 | 5,40 | aquaporin 10 [Source:HGNC Symbol;Acc:16029] |
| ZDHHC15 | 11,25 | 10,95 | 11,56 | 3,25 | 19,56 | zinc finger, DHHC-type containing 15 [Source:HGNC Symbol;Acc:20342] |
| AC010145.3 | 11,01 | 17,21 | 14,34 | 6,33 | 8,44 | [undefined] |
| GK | 10,80 | 15,25 | 9,30 | 4,06 | 12,54 | glycerol kinase [Source:HGNC Symbol;Acc:4289] |
| PBX1 | 10,64 | 19,67 | 12,29 | 6,85 | 9,21 | pre-B-cell leukemia homeobox 1 [Source:HGNC Symbol;Acc:8632] |
| ANKRD45 | 10,27 | 10,52 | 10,02 | 11,18 | 7,95 | ankyrin repeat domain 45 [Source:HGNC Symbol;Acc:24786] |
| DNER | 9,94 | 9,99 | 5,84 | 9,88 | 21,66 | delta/notch-like EGF repeat containing [Source:HGNC Symbol;Acc:24456] |
| VNN3 | 9,79 | 9,32 | 10,27 | 4,51 | 11,94 | vanin 3 [Source:HGNC Symbol;Acc:16431] |
| IL23R | 8,99 | 14,16 | 7,95 | 10,17 | 4,36 | interleukin 23 receptor [Source:HGNC Symbol;Acc:19100] |
| BCL6B | 8,61 | 10,02 | 8,91 | 5,37 | 8,33 | B-cell CLL/lymphoma 6, member B [Source:HGNC Symbol;Acc:1002] |
| FAM5C | 8,23 | 14,25 | 7,17 | 3,37 | 9,46 | family with sequence similarity 5, member C [Source:HGNC Symbol;Acc:22393] |
| GNGT1 | 8,08 | 9,86 | 6,62 | 2,95 | 12,16 | guanine nucleotide binding protein (G protein), gamma transducing activity polypeptide 1 [Source:HGNC Symbol;Acc:4411] |
| TPD52L3 | 7,75 | 9,50 | 11,56 | 4,22 | 6,33 | tumor protein D52-like 3 [Source:HGNC Symbol;Acc:23382] |
| CLCA3P | 7,52 | 7,37 | 7,67 | 5,25 | 11,86 | chloride channel accessory 3 (pseudogene) [Source:HGNC Symbol;Acc:2017] |
| CECR1 | 7,12 | 10,69 | 6,90 | 5,12 | 7,35 | cat eye syndrome chromosome region, candidate 1 [Source:HGNC Symbol;Acc:1839] |
| OPRK1 | 6,89 | 7,77 | 6,11 | 4,87 | 14,44 | opioid receptor, kappa 1 [Source:HGNC Symbol;Acc:8154] |
| CYSLTR2 | 6,60 | 4,23 | 5,88 | 10,21 | 7,40 | cysteinyl leukotriene receptor 2 [Source:HGNC Symbol;Acc:18274] |
| BAAT | 6,11 | 10,12 | 3,27 | 8,60 | 4,34 | bile acid CoA: amino acid N-acyltransferase (glycine N-choloyltransferase) [Source:HGNC Symbol;Acc:932] |
| DKKL1 | 5,96 | 4,72 | 7,57 | 5,92 | 6,00 | dickkopf-like 1 [Source:HGNC Symbol;Acc:16528] |
| CCDC62 | 5,89 | 8,91 | 5,63 | 4,93 | 6,16 | coiled-coil domain containing 62 [Source:HGNC Symbol;Acc:30723] |
| GAL3ST1 | 5,88 | 6,47 | 5,34 | 3,94 | 13,00 | galactose-3-O-sulfotransferase 1 [Source:HGNC Symbol;Acc:24240] |
| FAM107A | 5,64 | 8,17 | 4,85 | 4,10 | 6,57 | family with sequence similarity 107, member A [Source:HGNC Symbol;Acc:30827] |
| MECOM | 5,61 | 8,71 | 3,61 | 2,73 | 14,77 | MDS1 and EVI1 complex locus [Source:HGNC Symbol;Acc:3498] |
| C19orf30 | 5,45 | 7,27 | 2,38 | 5,59 | 5,31 | chromosome 19 open reading frame 30 [Source:HGNC Symbol;Acc:30049] |
| NPTX2 | 4,72 | 10,06 | 4,30 | 5,18 | 2,92 | neuronal pentraxin II [Source:HGNC Symbol;Acc:7953] |
| ATF7IP2 | 4,71 | 6,08 | 3,49 | 3,65 | 14,67 | activating transcription factor 7 interacting protein 2 [Source:HGNC Symbol;Acc:20397] |
| FSTL5 | 4,55 | 4,86 | 4,27 | 2,62 | 6,26 | follistatin-like 5 [Source:HGNC Symbol;Acc:21386] |
| CNGB1 | 4,52 | 5,07 | 3,88 | 4,04 | 8,04 | cyclic nucleotide gated channel beta 1 [Source:HGNC Symbol;Acc:2151] |
| LY6H | 4,47 | 7,08 | 3,14 | 5,13 | 3,90 | lymphocyte antigen 6 complex, locus H [Source:HGNC Symbol;Acc:6728] |
| SASH3 | 4,37 | 3,79 | 3,67 | 5,04 | 14,12 | SAM and SH3 domain containing 3 [Source:HGNC Symbol;Acc:15975] |
| SLC6A7 | 4,32 | 7,87 | 5,33 | 2,59 | 3,51 | solute carrier family 6 (neurotransmitter transporter, L-proline), member 7 [Source:HGNC Symbol;Acc:11054] |
| FAM98B | 4,09 | 3,45 | 4,86 | 2,06 | 15,05 | family with sequence similarity 98, member B [Source:HGNC Symbol;Acc:26773] |
| FGF12 | 4,02 | 7,10 | 2,87 | 5,64 | 2,21 | fibroblast growth factor 12 [Source:HGNC Symbol;Acc:3668] |
| RP11-159J16.1 | 3,95 | 6,47 | 2,36 | 3,14 | 4,95 | [undefined] |
| PDZK1IP1 | 3,94 | 2,35 | 4,65 | 3,33 | 5,45 | PDZK1 interacting protein 1 [Source:HGNC Symbol;Acc:16887] |
| PRSS21 | 3,85 | 4,56 | 3,25 | 11,07 | 2,30 | protease, serine, 21 (testisin) [Source:HGNC Symbol;Acc:9485] |
| DSTYK | 3,84 | 3,50 | 4,21 | 4,53 | 2,06 | dual serine/threonine and tyrosine protein kinase [Source:HGNC Symbol;Acc:29043] |
| AC011472.1 | 3,83 | 2,18 | 7,06 | 3,19 | 4,59 | Hepatocellular carcinoma-associated protein TD26 [Source:UniProtKB/Swiss-Prot;Acc:Q6UXH0] |
| CIITA | 3,69 | 4,89 | 2,79 | 2,48 | 5,79 | class II, major histocompatibility complex, transactivator [Source:HGNC Symbol;Acc:7067] |
| VIL1 | 3,65 | 3,72 | 3,59 | 2,15 | 5,12 | villin 1 [Source:HGNC Symbol;Acc:12690] |
| FAM123A | 3,65 | 4,10 | 7,33 | 3,24 | 2,47 | family with sequence similarity 123A [Source:HGNC Symbol;Acc:26360] |
| GABRB3 | 3,44 | 5,00 | 2,25 | 2,36 | 6,61 | gamma-aminobutyric acid (GABA) A receptor, beta 3 [Source:HGNC Symbol;Acc:4083] |
| FAM187B | 3,43 | 3,06 | 3,84 | 2,29 | 8,13 | family with sequence similarity 187, member B [Source:HGNC Symbol;Acc:26366] |
| RP3-527B10.1 | 3,34 | 5,31 | 3,45 | 3,23 | 2,08 | [undefined] |
| C4orf36 | 3,12 | 5,33 | 2,67 | 2,00 | 3,65 | chromosome 4 open reading frame 36 [Source:HGNC Symbol;Acc:28386] |
| KLF17 | 2,99 | 2,65 | 3,38 | 2,06 | 15,75 | Kruppel-like factor 17 [Source:HGNC Symbol;Acc:18830] |
| AC018692.1 | 2,85 | 8,56 | 2,37 | 2,28 | 3,42 | [undefined] |
| ZNF99 | 2,83 | 2,59 | 3,08 | 2,00 | 4,46 | zinc finger protein 99 [Source:HGNC Symbol;Acc:13175] |
| CYP1A1 | 2,54 | 2,85 | 3,67 | 2,07 | 2,26 | cytochrome P450, family 1, subfamily A, polypeptide 1 [Source:HGNC Symbol;Acc:2595] |
| NAV3 | 0,28 | 0,59 | 0,34 | 0,19 | 0,22 | neuron navigator 3 [Source:HGNC Symbol;Acc:15998] |
| NAV3 | 0,24 | 0,55 | 0,28 | 0,18 | 0,20 | neuron navigator 3 [Source:HGNC Symbol;Acc:15998] |
|  |  |  |  |  |  |  |
|  | Median fold change in all samples (red: >2, blue: <0.5) | Fold change in individual time points | | | |  |

Table 4**.**  *IL23R* and *NAV3* expression in colorectal cancer cell line CCL 248 compared to the normal colon cell line CRL 1541 as determined with qPCR. *NAV3* and reference gene *TBP* runs were performed in triplicate for each of the studied cell lines, with standard deviations for the crossing point (stdCp) varying from 0.03 to 0.37 for the NAV3 run, whereas for the *TBP* run it varied from 0.05 to 0.2, indicating high reproducibility of the experiments.

| Cell line | Normal colon cells  CRL-1541 | Colorectal cancer  CCL-248 |
| --- | --- | --- |
| *NAV3* status | Normal 2 copies (FISH) | Loss of *NAV3* in 9/10 metaphases |
| *NAV3/TBP* (normalized value of *NAV3* expression) | 40.3 | 0.7 |
| Percentage of *NAV3* expression compared to CRL-1541 | 100 | 1.7 |
| *IL23R/TBP* (normalized value of *IL23R* expression) | 438.5 | 1561.6 |
| Percentage of *IL23R* expression compared to CRL-1541 | 100 | 356,2 |
| *IL23R/NAV3* | 10.9 | 2230.8 |
| *IL23R/NAV3* expression  compared to CRL-1541 | 1 | 205.0 |
