# Supplementary material for: Potential role of a navigator gene NAV3 in colorectal cancer
Source: Br J Cancer. 2011 Dec 15;106(3):517–24. doi: 10.1038/bjc.2011.553 (PMC3273355; doi:10.1038/bjc.2011.553)
Supplement: Supplementary online material methods 1–4 [file bjc2011553x3.doc]

Supplementary online material, methods

**Supplementary method 1. Microsatellite instability analysis** Mononucleotide repeat markers BAT25 and BAT26 from the Bethesda panel (Boland et al., Cancer Res 58: 5248 – 5257, 1998) were used to determine the microsatellite instability (MSI) status, supplemented with five dinucleotide repeat markers (D12S1684, D12S326, D12S1708, D18S474, and D9S167) taken from our LOH panel. Given the specificity of mononucleotide repeat markers for high-degree MSI (Esemuede I et al., Ann Surg Oncol 17: 3370 – 3378, 2010), samples with two or more unstable markers, at least one of which was a mononucleotide repeat, were considered to have MSI whereas all other samples were regarded microsatellite-stable (MSS). In the present investigation, all MSI cases had both BAT25 and BAT26 unstable.

**Supplementary method 2. FISH probe labeling.** For analyzing patient samples, two bacterial artificial chromosome (BAC) clones specific to NAV3 DNA (RP11-36P3 and RP11-136F16; Research Genetics Inc., Huntsville, AL, USA) and the chromosome 12 centromere probe (pA12H8; American Type Cell Culture) were labeled with Alexa 594-5-dUTP and Alexa 488-5-dUTP (Invitrogen), respectively, using nick translation. BAC and centromere probes were mixed together with human COT-1 DNA (Invitrogen), precipitated and diluted into hybridization buffer (15 % w/v dextran sulphate, 70 % formamide in 2x SSC, pH 7.0).

For analyzing cell lines NAV3 specific BAC-probes (see above) were prepared and labeled with digoxigenin and centromere 12-specific probe was prepared and labeled with biotin or dUTP conjugated with fluorescein isothiocyanate (FITC) by nick translation as described previously (Karenko et al. 2005) or similarly with Diethylaminocoumarin - 5-dUTP (DEAC, Perkin Elmer Life and Analytical Sciences, Boston, MA, USA).

**FISH methods.** Nuclei slides were pretreated with 1 M sodium thiocyanate at +80°C for 5 minutes, washed three times with 2 x SSC, treated with 50 % glycerol, 0.1 x SSC at +90°C for 6 minutes, washed with 2 x SSC for 3 minutes and with distilled water three times for 2 minutes. Slides were digested with proteinase K (Sigma; 8μg/ml in 20 mM Tris-HCl, pH 7.5, 2 mM CaCl_2_) at +37°C for 8 minutes. After dehydration and air drying, probe mix was added, slides were denatured for 6 min at +85°C and hybridized for 48 hr at +37°C. Slides were washed three times with 1.5 M Urea, 0.1 x SSC at +47°C for 10 minutes, with 0.1 x SSC for 10 minutes at +47°C, three times with PBS, 0.1 % NP-40 at room temperature, rinsed with distilled water, air dried and mounted in Vectashield Mounting Medium with 4´,6-diamino-2 phenylindole dihydrochloride (DAPI; Vector).

FISH slides of patient samples were evaluated using Olympus BX51 microscope (Tokyo, Japan) equipped with a 60X oil immersion objective and a triple bandpass filter for simultaneous detection of Alexa488, Alexa594 and DAPI (Chroma Technology Corp., Brattleboro, VT, USA).

Conventional metaphase slides were prepared for FISH as previously described (Abdel-Rahman et al. 2001)**.** The hybridization of the probes and detection of digoxigenin-labeled NAV3-probes and biotin labeled centromere 12-specific probes were performed with avidin-FITC (Vector laboratories, Burlingame, CA, USA) and sheep anti-digoxigenin conjugated with rhodamine (Roche, Mannheim, Germany) as described previously (12). The armspecific MFISH was performed with XaCyte kit (Metasystems GmbH, Altlusheim, Germany) as recommended by the manufacturer). The hybridized metaphases were analyzed with epifluorescence microscope (Axioplan Imaging 2, Zeiss, GmbH, Jena, Germany equipped with a CCD-camera), and MFISH-program module (Isis, Metasystems, Altlussheim, Germany) either manually or using an automatic capturing facility (Metafer, Metasystems, Altlussheim, Germany).

**Supplementary method 3. LOH analysis.** DNA samples were first PCR amplified using primers rs1852464F 5’ CCTGCTATTTTCATCTTTCAAGC 3’ and rs1852464R 5’ GGCTGGGATGCTGTTTGAG 3’ to yield a 130 bp PCR fragment containing the A/G polymorphism. The PCR product was subsequently purified by Exonuclease I (10 U/μl) and SAP (Shrimp alkaline phosphatase, 2 U/μl) (ExoSAP-IT, Amersham Biosciences) and PCR Extension was performed using a fluorescently labeled extension primer 5’ GATGCTGTTTGAGCGCATCATGCTGGGCCC 3’ and nucleotide mix containing ddCTP. Extension products were 43 bp or 49 bp depending on whether G or A was present in the template and they were separated and results analyzed as previously described (13).

**Supplementary method 4. NAV3 siRNA transfection.** Normal colon cell lines were cultured in 6-well plates to 70% confluence, and thereafter transfected with 200 pmol of NAV3 siRNA pool or scrambled control siRNA (Dharmacon, IL, USA), using Dharmafect1 transfection reagent (Dharmacon). For the transfection of one 6-plate well, the siRNA was diluted to 1 µM in 100 µl of 1x siRNA buffer (received from manufacturer) and mixed with an equal amount of normal growth medium. Four microliters of transfection reagent was incubated for 5 minutes at room temperature with 196 µl of growth media. The siRNA dilution was added and incubated for 20 minutes at room temperature before addition to the cells in 1600 µl of growth media. Six hours post transfection, the transfection medium was replaced with normal growth medium. The viability of the cells was monitored 48 hours after transfection with trypan blue staining. Both cell types showed over 70% viability, and in addition, only adherent cells were used for RNA preparation.

**Supplementary method 5. Immunohistochemistry on tissue microarrays**. Immunohistochemistry was carried out by using the avidin-biotin-peroxidase complex tehnique (Vectastain Elite ABC kit [mouse IgG], Vector laboratories, Burlingame, CA, USA) with DAB as chromogenic substrate and Mayer’s hematoxylin as counterstain. Following standard deparaffinization, endogenous peroxidase activity was blocked in 3% H_2_O_2_ in PBS for 10 min and the sections were pretreated in a +95ºC water bath in citrate buffer (DakoCytomation, Glostrup, Denmark) for 20 min. The slides were then incubated with mouse monoclonal antibodies against IL23R (R&D MAB14001; diluted in  1:30, slides pretreated with 1% trypsin at 37C 30min) or against beta-catenin (Zymed CAT-5H10, Invitrogen, Carlsbad, CA 92008 U.S.A; diluted in 1:250, slide pretreatment at +95C for 30 min in DAKO Target Retrieval  Solution S1699, pH 6-6,2) at +4ºC overnight. DAB was used as colourigenic substrate for IL23R while VECTOR NovaRED substrate kit SK-4800 was used for beta-catenin. For NAV3 staining, the following antibody was used: HPA032111, Sigma-Aldrich, Chemie Gmbh, Munich, Germany, at dilution 1:300.
